# Supplementary material for: Characterizing changes in glucocorticoid receptor internalization in the fear circuit in an animal model of post traumatic stress disorder
Source: PLoS One. 2018 Dec 7;13(12):e0205144. doi: 10.1371/journal.pone.0205144 (PMC6286002; doi:10.1371/journal.pone.0205144)
Supplement: S1 Fig — (DOCX) [file pone.0205144.s001.docx]

**APPENDIX**

**Separating cytoplasmic from nuclear fraction in brain regions**

In this study, we used a methods described by Spencer et al., (2000) to separate cytoplasmic and nuclear fractions from dissected brain regions. To verify the efficacy of this separation technique, we performed western blot on dissected dorsal hippocampal sections and probed for β-tubulin (present only in the cytoplasm) and β-actin (present in the cytoplasm and nucleus). The results are shown in Figure S1A. β-tubulin was only observed in cytolasmic fraction, while β-actin was observed in cytoplasmic and nuclear fractions. These results demonstrated that we could extract cytoplasm and nuclear fractions from dissected brain regions using methods described in the manuscript.


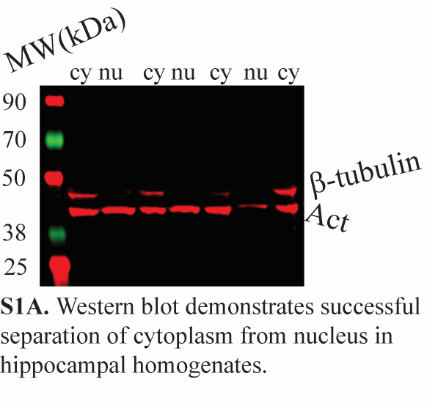


References

Spencer RL, Kalman BA, Cotter CS, Deak T. 2000. Discrimination between changes in glucocorticoid receptor expression and activation in rat brain using western blot analysis. *Brain Res* **868**: 275-286.
